# Pharyngoesophageal Obstruction on the Killian-Laimer Triangle by Zenker’s Diverticulum: Case Report and Clinical Significance

**DOI:** 10.14740/gr696w

**Published:** 2015-12-31

**Authors:** Tulio F. Leite, Carlos A. A. Chagas, Lucas A. S. Pires, Rafael Cisne, Marcio A. Babinski

**Affiliations:** a Department General Surgery, Santa Casa Hospital, Ribeirao Preto, SP, Brazil; b Department of Morphology, Biomedical Institute, Fluminense Federal University, Niteroi, RJ, Brazil

**Keywords:** Zenker’s diverticulum, Pharyngoesophageal diverticula, Dysphagia

## Abstract

Zenker’s diverticulum is a form of esophageal and pharyngeal obstruction located at the Killian-Laimer triangle. It is relatively common in elderly man (seventh or eighth decade of life), and its pathophysiology is not completely understood, albeit theories regarding dysfunction of the upper esophageal sphincter were reported. The main symptoms are dysphagia and odynophagia, but it can complicate to aspiration and perforation of the pharyngeal pouch; also, it can be asymptomatic. Diagnosis is provided through a barium esophagogram. Treatment can be performed through endoscopic surgery, diverticulopexy and myotomy of the cricopharyngeus muscle, although there is no consensus among surgeons regarding the treatment of choice. We report a case of pharyngeal obstruction due to Zenker’s diverticulum which caused massive weight loss in a 76-year-old male.

## Introduction

Zenker’s diverticulum is the most common form of pharyngeal and esophageal obstruction. It is caused by a loss of elasticity and muscle tone of the mucosae and submucosae layers of the esophagus. The diverticulum is located at the Killian-Laimer’s triangle [1, 2]. The Zenker’s diverticulum is rare, and it is found in the elderly, mostly at the sixth, seventh or eighth decade of life. It can be asymptomatic for weeks or years, but, in its symptomatic form, it presents with symptoms such as dysphagia and odynophagia [2]. The diagnosis for Zenker’s diverticulum is usually done through a barium esophagogram, but the diverticulum can also be diagnosed by an endoscopy [3]. The most common treatment for the Zenker’s diverticulum is endoscopic surgery, since it is minimally invasive and has a high success rate [4-6]. It is crucial to differentiate the Zenker’s diverticulum from the Killian-Jamieson diverticulum, considering the differences during surgical treatment [6, 7]. The current study reports a Zenker’s diverticulum in a 76-year-old male.

## Case Report

**Figure 1:**
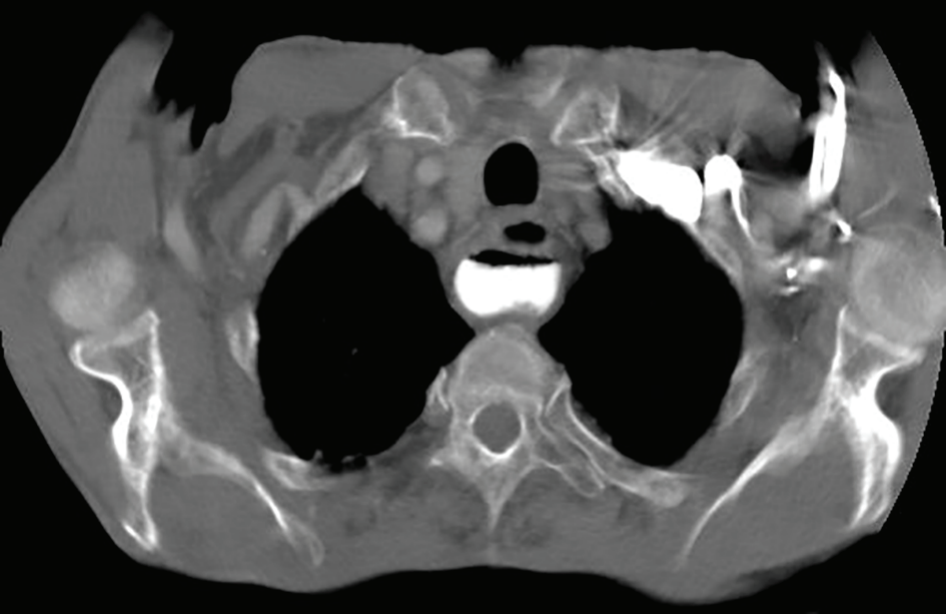
Thoracic CT scan showing the Zenker’s diverticulum.

**Figure 2:**
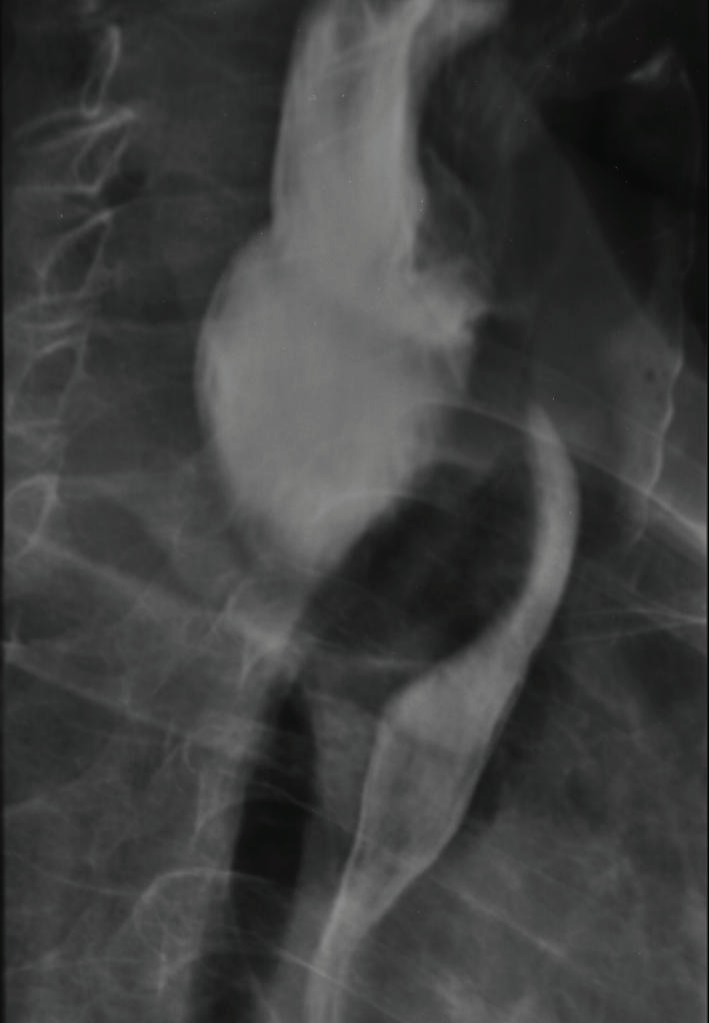
Esophagogram exhibiting extrinsic esophageal obstruction caused by the diverticulum.

**Figure 3:**
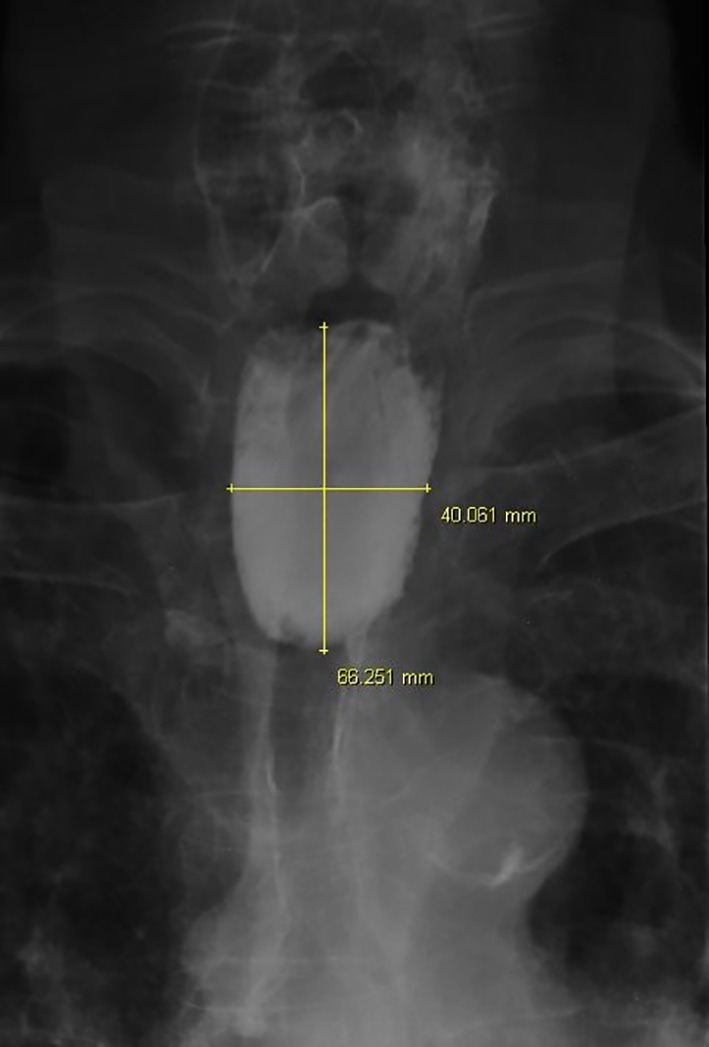
Anteroposterior esophagogram displaying the measures of the Zenker’s diverticulum.

A 76-year-old male patient presented with a history of progressive dysphagia for solids for a year, which evolved to dysphagia and odynophagia for liquids in 5 months. The patient was referred from a basic health unit to the General Surgery Service of “Santa Casa de Ribeirao Preto” with esophageal obstruction and suspicion of neoplasia. The patient had a slim figure, as he lost 20 kg in 5 months. His current weight was 39 kg, his height was 1.63 m and his BMI was 14.67 kg/m^2^. The patient confirmed smoking habits (two packs of cigarettes a day during 50 years) and claimed he was a social drinker; the patient also denied any comorbidity. He then went through an upper gastrointestinal endoscopy (UGE), and the endoscope only entered 22 cm below the upper dental arcade, which discarded the possibility of a biopsy, which was needed for the hypothesis of neoplasia. Thoracic and abdomen CT scan showed a thickening of the gastric walls, especially at the esophagogastric junction. The CT scan also revealed an abrupt straightening of the proximal third of the esophagus (Fig. 1), suggesting esophageal neoplasia. As he was unable to receive oral diet, we opted to provide his diet through a Witzel’s jejunostomy in order to improve his nutritional gains. The neoplasia hypothesis indicated the need to perform a biopsy to analyze the tissue, and if positive for neoplasia, refer the patient to the oncology service for neoadjuvant therapy. The patient, then, underwent through another UGE, but this time, the endoscope passed through the obstruction and identified the presence of a Zenker’s diverticulum. Next, he underwent through an esophagogram that identified a large esophageal diverticulum (Fig. 2, 3). Thus, the prognosis changed, and it indicated an endoscopic surgical repair of the Zenker’s diverticulum, as it is the standard procedure. Due to his low BMI and his nutritional conditions, it was opted to delay surgery.

## Discussion

The Zenker’s diverticulum, also known as pharyngoesophageal diverticulum or cricopharyngeus diverticulum, is described as an esophageal pseudodiverticulum, as its walls are composed by the mucosa and submucosa layers of the esophagus. It was described by Ludlow in 1769 and in 1878 by Friedrich Von Zenker [1, 2].

It occurs in 0.01-0.11% of the population, being more frequently encountered in the elderly, usually at the sixth, seventh or eighth decade of life. In the United Kingdom, the diverticulum has an annual incidence of two people for every 100,000. The Zenker’s diverticulum rarely affects individuals below 40 years old. The presence of a second diverticulum, albeit smaller, is verified in 1-2% of cases [2, 3, 8]. Despite being a rare clinical entity, the exact prevalence of this disease canot be accurately measured, considering the fact that the Zenker’s diverticulum is asymptomatic in numerous cases, thus, making the diagnosis inexistent. It occurs more often in men than in women, and the diverticulum can be associated with other diseases, such as Barret’s esophagus, hiatal hernia, achalasia, laryngocele, leiomyoma, polymyositis and esophageal stenosis [2, 6].

The Zenker’s diverticulum can cause mild symptoms, like foreign body sensation and sore throat, but it can also cause more intense symptoms, such as undigested food regurgitation, dysphagia, halitosis, odynophagia and significant weight loss. In severe cases, the diverticulum can lead to aspiration pneumonia, hemorrhage, vocal cords paralysis and tracheoesophageal fistula, but in some cases, it can also be asymptomatic. The diverticulum is also a predisposing factor for carcinoma, since it also causes chronic inflammation [1-3, 8].

The diverticulum commonly occupies the posterior esophageal wall at the left side of the neck, in a potentially weak area called Killian-Laimer’s triangle. This triangle is located between the oblique fibers of the thyropharyngeus muscle and the transverse fibers of the cricopharyngeus muscle. Those muscles are part of the inferior pharyngeal constrictor muscle and are innervated by the plexus pharyngeus of the vagus nerve. Nevertheless, there are other weak areas around the esophagus, such as the Killian-Jamieson’s area, located between the transverse fibers of the cricopharyngeus muscle and the longitudinal fibers of the esophageal wall [2, 9-13].

The pathophysiology of the disease is still in debate, but the most accepted hypothesis is that there is a high hypopharynx pressure on the muscles of the Killian-Laimer’s triangle, caused by fibrosis and motor dysfunction of the upper esophageal sphincter (UES), which can result in herniation of the esophagus’ mucosa and submucosa walls, forming the diverticulum. Other theories include cricopharyngeal spasms, congenital weakness of the area and gastroesophageal reflux [2, 3, 9-11, 14]. The UES is constituted by the posterior surface of the thyroid and cricoid cartilages, the cricopharyngeus muscle, the thyropharyngeus muscle and the cervical portion of the esophagus. The contribution of the cricopharyngeus muscle seems to be the most significant [3, 10, 13].

The Zenker’s diverticulum growth can be divided into four distinct stages: in the first stage, the diverticulum looks similarly to a 2 - 3 mm thorn, being only visible during the UES contraction. In the second stage, the diverticulum takes the shape of a 7 - 8 mm club, also only visible during the contraction phase of the UES. In the third stage, the diverticulum assumes the shape of a bag with caudal orientation and becomes bigger than 1 cm, being visible all the time. In the last stage, the diverticulum is similar to the third stage, but it causes esophageal compression [9]. Furthermore, the diverticulum is classified regarding its size: < 2 cm is classified as a small diverticulum, between 2 and 4 cm is classified as an intermediate diverticulum and 4 - 6 cm is classified as a large diverticulum [8].

Histologically, the Zenker’s diverticulum has a stratified squamous epithelium and its submucosa is surrounded by fibrosis. In some cases, squamous cell carcinoma or carcinoma *in situ* can be present (0.3-7% of cases); moreover, in rare and uncommon cases, an ulcerated diverticulum with the presence of lymphocytes and eosinophils can be found [1, 8].

The classic symptoms (dysphagia, odynophagia and excessive weight loss) associated with a palpable nodule at the cervical region (Boyce’s sign) and cervical borborygmi can provide the clinical diagnosis. The barium esophagogram or an upper digestive endoscopy can be performed to confirm the diagnosis. The esophagogastroduodenoscopy is not necessary to confirm the diagnosis, but it can be executed in order to rule out malignancy or the presence of associated diseases, such as hiatal hernia and gastroesophageal reflux [8, 12]. Oblique or lateral radiography can be used to identify the position and size of the diverticulum more accurately, and the ultrasound can be performed to rule out thyroid tumor [2, 8, 13]. It is extremely important to differentiate the Zenker’s diverticulum from the Killian-Jamieson’s diverticulum, as the latter needs a distinct surgical approach, since it is located at the Killian-Jamieson’s area, which has a more intimate relation to the recurrent laryngeal nerve, since he crosses the aortic arch and posteriorly ascends to the neck. Iatrogenic lesion or pathological lesion of these nerves can result in muscular function loss, hoarseness and dysphonia [7, 11-13].

Endoscopic surgery is the standard treatment for the Zenker’s diverticulum, since it is less invasive, faster and the fact that it does not need general anesthesia or hyperextension of the neck. But for the surgery to succeed, it is vital to have an adequate exposure of the area and the absence of prior relapses, if those factors exist, open surgery, diverticulectomy, diverticulopexy or cricopharyngeus myotomy, is advised. The cricopharyngeus myotomy is performed regardless of a diverticulectomy or diverticulopexy, as it avoids fistula formation caused by the excessive pressure at the area of the diverticulum and diminishes relapse rates. The choice of the procedure needs to be in accordance with the size of the diverticulum and the presence of comorbidities, such as Parkinson’s disease, which causes decreased pharyngeal contraction, making the cricopharyngeus myotomy ineffective. The most common risks (8-18% of cases) associated with open surgery are mediastinitis, recurrent laryngeal nerve lesion, pneumonia, hematoma, infection, and fistula formation, and the most common risks (4% of cases) associated with endoscopic procedures are hemorrhage and perforation, although they correspond directly to the surgeon’s ability [4-8, 12-14].

During the process of the diagnosis and treatment, it is necessary to evaluate the risk or the presence of malnourishment through BMI calculi and the evaluation of daily dietary habits associated to a recurrent weight loss. The weight loss occurs due to symptoms like dysphagia and odynophagia. Malnutrition is a factor that directly affects hospital stay and surgical outcome, considering that malnourished patients require more time to recover and are submitted to more risks during surgery [5]. We conclude that the Zenker’s diverticulum is a clinical entity that healthcare professionals should be aware since it has risks of malnourishment; furthermore, surgeons must have knowledge of the anatomical relations of the diverticulum and the risks associated with surgical intervention.
